# How do omission and commission errors of trait memory distrust relate to immediate and delayed suggestibility as measured by the Gudjonsson Suggestibility Scale?

**DOI:** 10.3389/fpsyg.2026.1774327

**Published:** 2026-03-09

**Authors:** Gisli Gudjonsson, Valeria Giostra, Monia Vagni

**Affiliations:** 1 Institute of Psychiatry, Psychology and Neuroscience, King’s College London, London, United Kingdom; 2 Centre for Education and Research in Forensic Psychology, The Department of Humanities, University of Urbino, Urbino, Italy; 3 Department of Philosophy, Social Sciences, Humanities and Education, University of Perugia, Perugia, Italy

**Keywords:** commission errors, confabulation, delayed suggestibility, immediate suggestibility, memory distrust, omission errors

## Abstract

The main purpose of the current study was to test the hypothesis that immediate and delayed suggestibility, measured by the Gudjonsson Suggestibility Scale (GSS 2), are more strongly associated with perceived susceptibility to commission errors in memory distrust than omission errors. There were 229 community participants, who had completed the GSS 2, and Memory Distrust Scale (MDS) and the Squire Subjective Memory Questionnaire (SSMQ). The MDS and the SSMQ measure people’s beliefs about their susceptibility to memory errors of commission and omission, respectively. There were several important findings. The main finding in common across the MDS and SSMQ was a significant negative relationship between GSS 2 memory recall and trait memory distrust. This was a robust finding and suggests that both commission and commission memory trust are associated with poorer objective memory recall. In contrast, only the MDS score was significantly correlated with GSS 2 confabulation, Yield 1, Yield 2, ‘direct explanation’ explanation resistant behavioural responses, and delayed suggestibility at 1 week follow-up. In contrast GSS 2 Shift correlated significantly with the SSMQ score but not the MDS score. The main implications of the findings are that commission errors, as measured by the MDS, are more associated with both immediate and delayed suggestibility than omission errors, and Shift with omission errors.

## 1 Introduction

The main objective of the present study is to examine the relationship between immediate and delayed interrogative suggestibility, as measured by the Gudjonsson Suggestibility Scale (GSS-2; Gudjonsson, 1987, 1997; Vagni et al., 2015), and both trait-level commission and omission errors, as assessed by the Memory Distrust Scale (MDS; Nash et al., 2023) and the Squire Subjective Memory Questionnaire (SSMQ; Van Bergen et al., 2010a, 2010b), respectively. The inclusion of both the MDS and the SSMQ is theoretically and methodologically motivated in order to distinguish between qualitatively different forms of subjective memory vulnerability in relation to immediate and delayed suggestibility. In this respect, the current study adopts a distinctive and integrative approach.

The position taken is that, whilst both commission and omission errors are relevant to memory distrust, commission errors constitute the more central and diagnostically informative indicator of immediate and delayed suggestibility, particularly in forensic contexts where acceptance of misleading information and source-monitoring failures are of primary concern (Gudjonsson, 2018).

### 1.1 Memory Distrust Syndrome

Based on their experience of real-life criminal cases, Gudjonsson and MacKeith (1982, p. 265) introduced the term Memory Distrust Syndrome to describe “some persons tendencies to be persuaded that they might have committed a crime because they do not trust their own memory due to previous memory impairment.” Based on evaluation of more real-life cases, Gudjonsson (2003, p. 196) provided a definition of Memory Distrust Syndrome as “a condition where people develop profound distrust of their memory recollection, as a result of which they are particularly susceptible to relying on external cues and suggestions.” According to Van Bergen (2011), Gudjonsson and MacKeith (1982) introduced the concept of memory distrust into scientific literature, leading to a series of influential experimental studies (Van Bergen, 2011; Van Bergen et al., 2009, 2010a, 2010b).

What differentiates Memory Distrust Syndrome from simple memory distrust is that the associated symptoms (i.e., distrust in the accuracy, completeness and reliability of memory, and susceptibility to external influence) are profound and impact negatively on the person’s ability to give reliable statements during interrogation (Gudjonsson, 2018). Therefore, it may have more direct links to false internalised false confessions than simple memory distrust, which in a mild form is common in everyday life and may not necessarily interfere with normal functioning. When memory distrust becomes pronounced, it can interfere with normal functioning (Schacter, 2001).

In his extensive experience, Gudjonsson (2003) argued that Memory Distrust Syndrome was associated with two distinct conditions. First, suspects had no recollection of committing the offence due to amnesia or alcohol induced memory problems. Secondly, suspects had a recollection of not committing the crime, but their trust in their recollection was undermined by the way police interrogated them (Gudjonsson, 2017), leading to a confused mental state (Gudjonsson et al., 2021). Both conditions of profound memory distrust are characterised by commission errors (i.e., accepting and producing erroneous information), which typically involves a five-stage process comprised of a trigger, plausibility, acceptance, reconstruction, and resolution (Gudjonsson et al., 2014). In contrast, omission errors (i.e., failure to recall salient material) are less directly relevant to Memory Distrust Syndrome in police interrogation settings, although they may be a contributory factor (e.g., failure to provide an alibi without access to a diary due to memory decay and confusion elicited by her arrest, which made police suspicious – see the case of Carole Richardson, one of the ‘Guildford Four’ wrongly convicted for terrorist offences; Gudjonsson, 2003, p. 451).

Schacter (2001) provides a good distinction between omission and commission memory errors, the former being more associated with forgetting (referred to as ‘Transience’, ‘Absent-mindedness’, and ‘Blocking’), and commission errors, which are more akin to ‘Misattribution’ (i.e., source monitoring problems), memory distortions, confabulation, and suggestibility. Self-induced bias is also relevant in some cases of internalised false confessions (Gudjonsson et al., 1999) and forms a part of the acceptance of misleading information (Gudjonsson, 2003).

### 1.2 Interrogative suggestibility

Interrogative suggestibility is most pronounced in individuals who experience uncertainty about their own memory and recall ability, who hold expectations of success, and who display high levels of interpersonal trust. These characteristics form the core of the Gudjonsson and Clark (1986) interrogative suggestibility model and are particularly influential during custodial questioning when interrogators employ leading questions and exert social and emotional pressure on the interviewee.

To assess individual vulnerability to suggestive influence in an interrogation context, Gudjonsson developed and validated a standardised psychometric instrument: the Gudjonsson Suggestibility Scale (GSS 1 and GSS2; Gudjonsson, 1987). The GSS2 operationalises the principal components of *intermediate suggestibility* through three indices:

(1) Yield 1 – the tendency to accept misleading information embedded in leading questions;
(2) Shift – the tendency to change previously given answers following negative feedback via psychosocial pressure;
(3) Yield 2 – a second measure of acceptance of misleading information, obtained after the administration of negative feedback.

In subsequent developments, Vagni et al. (2015) and Gudjonsson et al. (2016, 2024) extended the standard GSS procedure by incorporating a *delayed suggestibility* measure. This extension involves assessing delayed recall after a one-week interval, allowing for the detection of post-event information effects arising from misleading suggestions introduced during the original suggestive interview. At follow-up, delayed suggestibility is measured during free recall of the GSS 2 narrative and reflects the extent to which participants have incorporated misleading post-event information derived from the Yield questions into their recollection.

There is an important distinction between delayed suggestibility and confabulation as measured by the Gudjonsson Suggestibility Scales. Delayed suggestibility reflects the incorporation of *external misleading information* into memory, whereas confabulation reflects the *internal generation* of false information in the absence of external suggestion.

In the first study of the Gudjonsson Suggestibility Scale (GSS), Gudjonsson (1983) found a significant overall negative correlation of Yield 1 (*r* = −0.29) and Yield 2 (*r* = −0.30) with self-reported confidence of normal participants in their performance regarding answers on the Yield items. This provided the first empirical link between confidence in memory and GSS Yield replies before and after negative feedback. In contrast, Shift replies were not significantly correlated with confidence in memory, suggesting a psychosocial rather than a memory confidence mechanism. It was the type of effect that negative feedback had on the subsequent answers that are important rather than Shift per se. The implication is that yielding to misleading questions (i.e., commission errors) is associated with lack of memory confidence and memory distrust.

Van Bergen (2011) makes an important point that memory distrust elicited by the administration of the GSS has both trait and state characteristics. Yield is said to be driven by trait memory distrust but Shift by state memory distrust (Gudjonsson, 2003). The implication is that trait questionnaires of memory distrust are more associated with Yield 1 and Yield 2 answers on the GSS than Shift.

The main purpose of the current study is to test the hypothesis that immediate and delayed suggestibility are more strongly associated with trait commission errors in memory distrust, as measured by the Nash et al. (2023) Memory Distrust Scale, than by omission errors measured by the Squire Subjective Memory Questionnaire (SSMQ; Van Bergen et al., 2008, 2009, 2010a, 2010b).

### 1.3 Commission and omission errors (MDS and SSMQ)

Memory Distrust Scale (MDS) and the Subjective Self-Memory Questionnaire (SSMQ) are self-report instruments that assess individuals’ beliefs about their susceptibility to memory errors of *commission* and *omission*, respectively. Unlike measures that focus primarily on fear of forgetting or memory lapses (errors of omission), the MDS specifically assesses the extent to which individuals believe they are prone to creating false memories or recalling events that did not occur.

The MDS has been empirically associated with interrogative suggestibility and has demonstrated good reliability, supporting its potential utility in forensic contexts. In contrast, the SSMQ assesses concerns about forgetting factual information and reflects general confidence in everyday memory functioning.

Vulnerability to both forgetting and false memory creation has important implications for individuals exposed to suggestive influences, particularly within forensic and investigative settings. The interaction between these two forms of memory self-belief—commission- and omission-related—warrants further systematic investigation.

Importantly, assessment of vulnerability in forensic contexts should not be limited to self-reported memory confidence and suggestibility alone. It should also incorporate *confabulation*, as an objective indicator of memory distortion and fabrication, and *compliance*, defined as the tendency to comply with the requests or demands of others in order to gain immediate advantage (e.g., terminating an interrogation) or to avoid conflict, even in the absence of private agreement. Confabulation can be indexed within the Gudjonsson Suggestibility Scales (GSS 1 and GSS2; Gudjonsson, 1997), while compliance is measured using the Gudjonsson Compliance Scale (GCS) (Gudjonsson, 1989).

Some previous studies have highlighted the correlation between memory distrust and suggestibility as measured with the GSS 2. Van Bergen et al., 2010a, 2010b found that memory distrust, as measured by SSMQ, was positively related to compliance as measured by the GCS; Gudjonsson Compliance Scale (Gudjonsson, 1989, 1997), although a significant correlation (*r* = −0.33) was only found in a community sample. In a different study, Van Bergen et al. (2009) found that SSMQ correlated negatively with compliance (*r* = −0.22), positively with immediate (*r* = 0.30) and delayed (*r* = 0.30) recall on the Gudjonsson Suggestibility (GSS 1), whilst there was no significant relationship found with GSS 1 Yield 1, Yield 2, Shift and Total Suggestibility. Nash et al. (2023) found a significant relationship between the Memory Distrust Scale and compliance (medium effect size).

We investigate the relationship between the MDS and the SSMQ with immediate and delayed recall on the GSS 2, which we predict is significant on both memory distrust measures (Nash et al., 2023). In contrast, we predict that confabulation in immediate and delayed recall is only significantly correlated with commission errors.

### 1.4 Suggestibility and perceived susceptibility to errors

The degree of confidence or uncertainty with which an individual responds to a leading question reflects both *mnemonic* and *social* components. The social component, as outlined above, involves a tendency to accept misleading information despite the interviewee not being internally convinced of its accuracy. This response style may arise from *susceptibility to suggestions*, *compliance*, or their interaction.

Confidence in one’s memory is also closely linked to *source monitoring ability*. Within the source monitoring framework developed by Johnson and Raye (1998), resistance to leading questions can take qualitatively different forms. Simple negative responses (e.g., “no”) are associated with a higher level of source monitoring than acquiescent responses. Individuals with particularly strong source monitoring skills may not only reject a misleading suggestion but also provide additional details or explicit justifications (e.g., “There was no such element in the story”). This more elaborated form of resistance has been termed *Direct Explanation* answers (Gudjonsson et al., 2021, 2022).

A less secure form of resistance involves expressions of uncertainty, such as “I don’t know.” Although these responses technically reject the misleading premise, they reflect weaker anchoring to one’s own memory and greater epistemic uncertainty.

Gudjonsson et al. (2021, 2022) empirically validated these three qualitatively distinct resistant responses (i.e., ‘Direct Explanation’, ‘No’, and ‘Do not Know’ replies) and formalised them within the Resistant Behavioural Responses (RBR) model (Gudjonsson & Clark, 1986). All three response types constitute rejection of the leading question. However, they differ meaningfully in their protective value. Direct Explanation and No responses are associated with greater resistance to suggestibility, whereas Do not Know responses are linked to increased vulnerability, particularly to Yield and Shift following negative feedback.

Despite their clear conceptual overlap, the relationship between the RBR model and memory distrust—including uncertainty in source monitoring and confidence in one’s own recollections—has not yet been systematically investigated. This represents an important gap in the literature, with direct implications for understanding individual vulnerability to suggestion in forensic interviewing contexts.

In addition to suggestibility, in this present study we investigate the relationship between different types of resistant behavioural responses (RBRs) and trait memory distrust regarding commission errors. Based on our previous research (Gudjonsson et al., 2022, 2024; Gudjonsson and Young, 2021) ‘direct explanation’ (DE) answers are most likely to be negatively correlated with memory distrust involving commission errors.

To the best of our knowledge, no study has investigated the relationship between the Memory Distrust Scale, compliance and immediate and delayed suggestibility using metric instruments with high construct validity. The two main relevant findings from the Nash et al. (2023) study are as follows. First, the MDS and the SSMQ scores are significantly correlated (*r* = − 0.46) suggesting that they are measuring similar underlying processes. Secondly, the MDS scores correlated significantly with compliance as measured by the Gudjonsson Compliance Scale (GCS; *r* = 0.32), whereas the correlation of the SSMQ score was negatively correlated with GCS compliance (*r* = −0.30). This is expected as the two measures are scored in reverse (i.e., a high score on the MDS and a negative score on the SMMQ).

*Hypotheses*.

The main hypotheses tested in the current study are as follows:

(1) Scores on the SSMQ and MDS are modestly and negatively correlated (i.e., small - medium effect size).
(2) Both the MDS and the SSMQ are significantly correlated with immediate and delayed memory on the Gudjonsson Suggestibility Scale.
(3) The GSS 2 confabulation scores on the GSS 2 will be positively correlated with the MDS but not with the SSMQ.
(4) The SSMQ and MDS scores will correlate both with immediate and delayed suggestibility.^1^
(5) Regarding resistant behavioural responses (RBRs) on the GSS 2, the MDS score will be most strongly associated, negatively, with ‘direct explanation’ (DE) answers.
(6) The MDS and SSMQ correlate significantly with compliance.

## 2 Methods

### 2.1 Participants

The study recruited 229 participants from the general population (mean age 28.54; SD = 12.70; min-max = 19–57); 170 participants (74.2%) were female. Participants met the inclusion criteria (a) understanding of the Italian language; (b) completing all the questions of the various instruments, excluding the missing ones; (c) immediate recall on the GSS 2 text within two standard deviation of the mean; and (d) absence of intellectual disabilities or other deficits that may limit reading and understanding of verbal items. (The study was limited to a self-declaration of intellectual disability to exclude failure to understand the questions due to intellectual deficit.)

The ±2 standard deviations (SDs) are often used to exclude *extreme scores* (often referred to as ‘outliers’) because, under a normal (Gaussian) distribution, about 95% of observations fall within ±2 SDs of the mean. Scores beyond that range are statistically *unusual* and more likely to reflect measurement error, data entry error, non-representative responding, or a qualitatively different process rather than the phenomenon of interest (Osborne and Overbay, 2004).

The sample was collected by convenience and on a voluntary basis without remuneration. From the initial sample, 25 participants were excluded because some had an immediate recall of the GSS2 below 2 SD and because they had not completed the second section after a week. The 229 participants included in the study met the inclusion criteria.

### 2.2 Instruments

#### 2.2.1 GSS 2

The GSS 2 (Gudjonsson Suggestibility Scale – Gudjonsson, 1987, 1997) consists of asking for an immediate recall of a story read. In Immediate Recall, the number of recalled items and any confabulations are counted, which are elements of distortion and fabrication that people can add to the original stimulus (Clare and Gudjonsson, 1993). After approximately 40–50 min, the participants are subjected to the administration of 15 leading questions and 5 neutral questions. The participant is given negative feedback, indicating that they made several mistakes and should try to do better, after which the 20 questions are repeated. The number of leading questions accepted at both the first and second interviews provides the Yield 1 and Yield 2 scores, respectively. The number of modified answers, both to neutral and leading questions, after negative feedback provides the Shift score.

In contrast to the standard procedure, the procedure of Gudjonsson et al. (2016) was followed, which consists in asking for a delayed recall of the story after 1 week, which provides the delayed recall score and confabulations as commission errors on delayed recall. The amount of post-event information from the leading questions provides the total delayed suggestibility score.

Confabulation (i.e., self-induced distortions and fabrications added together give the score of confabulations to the immediate memory) can also be measured (Gudjonsson, 1997), which differs from delayed suggestibility (Gudjonsson et al., 2024; Vagni et al., 2021).

Additionally, Resistant Behavioural Responses (RBR; Gudjonsson et al., 2021, 2022; Gudjonsson and Young, 2021) that make up Yield 1 and Yield 2 can be measured. Simple negative responses that reject the suggestive question are NO 1 and NO 2, respectively. Responses that firmly reject the misleading question by a comment like ‘it wasn’t mentioned in the story” (i.e., good source monitoring) are referred to as ‘direct explanation’ (DE 1 and DE 2, respectively). Dubitative responses of ‘I don’t know’ or ‘I’m not sure’ are referred to as DK 1 and DK 2, respectively.

#### 2.2.2 GCS

The Gudjonsson Compliance Scale (Gudjonsson, 1989, 1997) is a questionnaire of 20 items with true/false answers that detects compliance according to two main factors: The avoidance of conflict and confrontation and the desire to please others (see Hang et al., 2024, for a recent comprehensive validation study).

#### 2.2.3 MDS

The Memory Distrust Scale (Nash et al., 2023) is a 20-item self-report scale used to assess participants’ self-reported tendency to distrust their memories because of commission errors they may commit (misremembering and false remembering). The response mode includes a 1–7 Likert scale to express perception or agreement from 1 = strongly disagree to 7 = strongly agree. Since this is the first time that the MDS has been administered to an Italian sample, the Cronbach reliability was calculated and showed a good internal consistency (*α* = 0.91). The higher the score the greater the memory distrust.

The MDS is a trait-level measure designed to assess individuals’ distrust in their own memory and their propensity to defer to external sources when recalling or evaluating past events. This form of epistemic vulnerability is closely associated with commission errors and increased susceptibility to misleading information and interrogative suggestibility. This makes it particularly relevant to the present study.

#### 2.2.4 SSMQ

The Squire Subjective Memory Questionnaire (Van Bergen et al., 2010a, 2010b) is an 18-item questionnaire in which participants rate their memory functioning indicating for each item the perception of effectiveness with answer options ranging from ‘worse than ever before’ (−4) to ‘better than ever before’ (+4). The final score can go from −72 to +72. The internal consistency of items in the current study was good (*α* = 0.87). Negative scores indicate greater memory distrust.

The SSMQ is a trait-level measure of subjective memory functioning, designed to assess individuals’ perceived everyday memory ability and confidence. It indexes omission-oriented memory complaints rather than susceptibility to commission errors or interrogative suggestibility. It captures metamemory—that is, beliefs, appraisals, and self-monitoring of memory—rather than actual recall accuracy.

The Polish adaptation of the SSMQ (Kuczek et al., 2018) demonstrated satisfactory internal consistency, construct validity, and test–retest reliability. This finding indicates that translation into another language does not compromise the psychometric integrity of the instrument, which is directly relevant to the present study.

### 2.3 Procedure

In a preliminary phase, we translated the MDS and MSSQ measures into Italian under the supervision of a cultural mediator to ensure the translated items retained their original meaning (see appendices A2 and A3, respectively). Some subjects were asked to read the items and indicate whether they found them comprehensible.

All participants completed the same instruments and met in two sessions 1 week apart. In the first session, immediate suggestibility was assessed by reading the GSS 2 story, requiring immediate recall. After a latency period of approximately 40–50 min, participants were subjected to the suggestive interview according to the procedure of Gudjonsson et al. (2016). After the interview, participants filled out the GCS and approximately half of the sample (116 participants; 50.7%) filled out the MDS and SSMQ questionnaires to measure memory distrust.

We administered MDS and SSMQ in different sessions to half the sample to explore whether there was an order effect and whether the administration of GSS 2 led to an overall impact on memory distrust at 1 week follow-up (see Van Bergen et al., 2008, for a relevant study).

After 1 week, all participants were asked to recall the GSS 2 narrative, providing delayed suggestibility as well as delayed confabulation (Gudjonsson et al., 2016; Vagni et al., 2015). The remaining half of the sample (113 participants, 49.3%) filled out the MDS and SSMQ questionnaires that they had not completed in the first session.

The study conformed to all ethical guidelines for research with human participants and followed the Declaration of Helsinki. The informed consent was signed before the inclusion the participants in the study, and it contained information on the objective of the study, methods of conduct, anonymity, and information on the conservation of sensitive data. The study was approved by the bioethics committee of the University of Perugia (n.19/2025).

### 2.4 Analytical strategy

Principal component analyses were conducted for both MDS and SSMQ.

Cronbach’s alpha coefficients and McDonald’s omega were used to measures the internal consistency of the MDS and SSMQ items. Cronbach’s alpha quantifies the level of agreement on a standardised 0 to 1 scale (Taber, 2018). *t*-test comparisons were conducted between participants who completed MDS and SSMQ at the first and second sessions. To test the potential influence of the order of the instruments, a comparison of the correlations between MDS and SSMQ and GSS 2 scores were performed, obtaining z-scores (Fisher).

Pearson correlations were conducted between the GSS 2, GCS, MDS and SSMQ scores. Hierarchical linear regression models were performed to investigate which independent variables between best predicted immediate and delayed suggestibility scores, respectively. For immediate suggestibility, separate regressions were performed for Yield 1, Yield 2, and Shift, which were the dependent variables. Age, sex, immediate recall and confabulation on immediate recall were entered in Step 1. The GCS, MDS and SSMQ scores were added in Step 2.

The procedure was repeated for delayed suggestibility as the dependent variable, but two separate models were analysed with independent variables from GSS 2. In Model 1, age, sex, immediate recall and confabulation on immediate recall were entered in Step 1. NO 1, DE 1, DK 1, GCS, MDS and SSMQ scores were added in Step 2. In Model 2, age, sex, delayed recall and confabulation on delayed recall were entered in Step 1. NO 2, DE 2, and DK 2, Shift, GCS, MDS, SSMQ, and Shift were entered in Step 2.

## 3 Results

### 3.1 Preliminary analyses

The Principal Component Analysis was conducted on MDS performing the Kaiser-Meyer-Olkin (KMO) test and Bartlett’s test of sphericity and considering the scree plot and analysing the eigenvalues of a solution that included 1 to 10 factors, following the Kaiser-Guttman criterion, and then extracting the factors with eigenvalues greater than 1.0. Subsequently, the factors identified in the PCA were inspected at the item level and their internal validity was assessed using the Cronbach coefficient, McDonald’s omega, and item-score correlation. The PCA supported a single-factor structure. The KMO test revealed an overall value of 0.91 and Bartlett’s test of sphericity was significant, *χ*^2^ (190) = 2,218 *p* < 0.001. The scree plot analysis supports a single factor solution, with items saturating with values ≥0.500. The variance is 34.7%. The internal reliability shows a good fit in order to Cronbach coefficient (*α* = 0.91) and McDonald’s Omega (*U* = 0.91). The items had an intercorrelation between 0.329 and 0.660.

The Principal Component Analysis was replicated for SSMQ performing the Kaiser-Meyer-Olkin (KMO) test and Bartlett’s test of sphericity and considering the scree plot and analysing the eigenvalues of a solution that included 1 to 10 factors, following the Kaiser-Guttman criterion, and then extracting the factors with eigenvalues greater than 1.0. Subsequently, the factors identified in the PCA were inspected at the item level and their internal validity was assessed using the Cronbach coefficient, McDonald’s omega, and item-score correlation. The PCA supported a single-factor structure. The KMO test revealed an overall value of 0.82 and Bartlett’s test of sphericity was significant, *χ*^2^ (120) = 1,016 *p* < 0.001. The scree plot analysis supports a single factor solution, with items saturating with values ≥0.300. The variance is 28.9%. The internal reliability shows a good fit in order to Cronbach coefficient (*α* = 0.87) and McDonald’s Omega (*U* = 0.88). The items had an intercorrelation between 0.337 and 0.653.

Subsequently comparisons were made for testing order effects between-group differences for the results of the MDS and SSMQ questionnaires completed during the first and second sessions. Importantly, *t*-test comparisons did not reveal any significant differences between the two sessions (MDS1: mean = 57.29; SD = 16.60; MDS2: mean = 54.97; SD = 18.60; *t* = 0.996; p n.s.; SSMQ1: mean = 21.04; SD = 17.17; SSMQ2: mean = 21.13; SD = 16.42; *t* = −0.041; p n.s.). This shows that there were no significant order effects.

To test the potential influence of the order of the instruments, a comparison of the correlations between the scores of the MDS and SSMQ and GSS2 scores (Yield 1; Yield 2; Shift; Total Suggestibility; and Delayed Suggestibility) were performed, obtaining z-scores (Fisher). The analyses did not show significance, excluding the presence of significant effects related to the order of administration.

Pearson correlations of the various GSS 2 measures with the GCS, Memory Distrust Scale, and SSMQ are shown in Table 1. Correlations significant at *p* < 0.05 are not shown to avoid inflation of results due to multiple comparisons.

**Table 1:** Pearson correlations of the GSS 2 measures with the Memory Distrust and SSMQ scores (*N* = 229).

GSS2	GCS	MDS	SSMQ
Immediate recall	−0.076	−0.197*	0.261**
Confabulation on immediate recall	0.069	0.129	−0.072
Delayed recall	0.004	−0.196*	0.243*
Confabulation on DR	0.125	0.195*	0.029
Yield 1	0.251**	0.335**	0.021
Yield 2	0.353**	0.304**	−0.028
Shift	0.172*	0.097	−0.212*
Total suggestibility	0.277**	0.294**	−0.107
Delayed suggestibility	−0.033	0.326**	0.037
RBRs			
NO 1	0.032	0.008	−0.054
DE 1	−0.184*	−0.211*	0.098
DK 1	−0.051	−0.079	−0.121
NO 2	−0.092	0.024	0.026
DE 2	−0.187*	−0.228*	0.077
DK 2	−0.102	−0.098	−0.100
MDS	0.110	-	−0.160
SSMQ	0.011	−0.160	-

*p < 0.01; **p < 0.001. Correlations significant at *p* < 0.05 are not shown to avoid inflation of results due to multiple comparisons.

GCS, Gudjonsson Compliance Scale; MDS, Memory Distrust Scale; SSMQ, Squire Subjective Memory Questionnaire; GSS2, Gudjonsson Suggestibility Scale 2; NO 1 and 2, simple No answers; DE 1 and 2, Direct Explanation answers; DK 1 and 2, Do not Know answers.

Both immediate and delayed recall correlated significantly with the Memory Distrust Scale and the SSMQ, but the effect sizes were small.

The Memory Distrust Scale showed significant correlations with GSS 2 Yield 1, Yield 2 scores and Direct Explanation answers (DE 1 and DE 2; small effect size), but not with Shift. In contrast, SSMQ correlated significantly with Shift (small effect size). There was a significant negative correlation between MDS and SSMQ as predicted (small effect size). The GCS showed positive correlations with all immediate suggestibility scores and negative correlations with Direct Explanation responses (small to medium effect sizes). GCS scores did not correlate significantly with either MDS or SSMQ scores.

Table 2 shows the outcome of the two Steps in the hierarchical regression models for Yield 1, Yield 2 and Shift. The variables in Step 2 added 12.6, 15.1, and 5.7% to the variance in Yield 1, Yield 2, and Shift, respectively. GCS and MDS were the best predictors of Yield 1 and Yield 2, and SSMQ of Shift.

**Table 2:** Hierarchical linear regression models of the age, sex, immediate recall, confabulation, GCS, MDS, and SSMQ on immediate suggestibility scores (*n* = 229).

Predictor	Yield 1		Yield 2		Shift		Tolerance/VIF	
Step 1	*B*	*β*	*B*	*β*	*B*	*β*		
Intercept	3.85		4.90		5.87			
Age	0.02	0.07	0.01	0.04	−0.02	−0.09	0.89	1.12
Sex	1.09	0.16*	1.44	0.17**	0.30	0.05	0.98	1.00
Immediate recall	−0.14	−0.22**	−0.16	−0.22**	−0.09	−0.19*	0.83	1.20
Confabulation IR	0.12	0.06	0.14	0.06	0.04	0.02	0.92	1.08
	*R*^2^ = 0.101 *F* = 6.229***		*R*^2^ = 0.094 *F* = 5.777***		*R*^2^ = 036 *F* = 2.087			

Predictor	Yield 1		Yield 2		Shift		Tolerance/VIF	
Step 2	*B*	*β*	*B*	*β*	*B*	*β*		
Intercept	−0.68		−0.59		5.19			
Age	0.02	0.08	0.01	0.04	−0.02	−0.09	0.89	1.12
Sex	0.90	0.11	1.11	0.13	0.15	0.03	0.98	1.02
Immediate recall	−0.12	−0.19**	−0.13	−0.17*	−0.06	−0.13	0.78	1.28
Confabulation IR	0.05	0.02	0.05	0.02	0.02	0.01	0.92	1.09
GCS	0.14	0.19**	0.26	0.30***	0.09	0.16*	0.97	1.03
MDS	0.05	0.28***	0.05	0.23***	0.01	0.02	93	1.08
SSMQ	0.02	0.12*	0.01	0.06	−0.03	−0.19**	0.93	1.08
	*R*^2^ = 0.227 Δ*R^2^* = 0.126*** F = 9.177***		*R*^2^ = 0.245 Δ*R*^2^ = 0.151*** F = 10.164***		*R*^2^ = 0.093 Δ*R*^2^ = 0.057** F = 3.213**			

Sex: 1, male; 2, female; Gudjonsson Compliance Scale; MDS, Memory Distrust Scale, SSMQ, Squire Subjective Memory Questionnaire. *p < 0.05; **p < 0.01; ***p < 0.001.

Table 3 shows the outcome of the two Steps in the hierarchical regression models for delayed suggestibility. The variables in Step 2 added 15.5 and 12.0% to the variance in delayed suggestibility prior to (Model 1) and after negative feedback (Model 2). All RBRs added significantly to the variance in delayed suggestibility, as well as the GCS and MDS. The main contributory independent variables in Model 2 were consistent with those in Model 1, although less marked. Shift did not make a significant contribution to the variance in delayed suggestibility, but SSMQ did.

**Table 3:** Hierarchical linear regression models of the age, sex, immediate and delayed recall, confabulation on immediate and delayed recall; RBRs; GCS, MDS and SSMQ on delayed suggestibility (*n* = 229).

Predictor	Delayed suggestibility						
	Model 1				Model 2		
Step 1	*B*	*β*	VIF	Step 1	*B*	*β*	VIF
Intercept	0.84			Intercept	0.74		
Age	0.01	0.01	1.12	Age	0.01	0.03	1.11
Sex	0.12	0.07	1.00	Sex	0.09	0.05	1.00
Immediate recall	−0.04	−0.24**	1.23	Delayed recall	−0.04	−0.28***	1.12
Confabulation on IR	0.08	0.15*	1.11	Confabulation on DR	0.08	0.15*	1.02
	*R*^2^ = 0.109 *F* = 5.674***				*R*^2^ = 0.119 *F* = 6.223***		
	

Predictor	Delayed suggestibility						
	Model 1				Model 2		
Step 2	*B*	*β*	VIF	Step 2	*B*	*β*	VIF
Intercept	1.22			Intercept	0.86		
Age	0.01	0.02	1.17	Age	0.01	0.02	1.13
Sex	0.09	0.05	1.02	Sex	0.07	0.04	1.03
Immediate recall	−0.03	−0.17*	1.40	Delayed recall	−0.03	−0.19*	1.43
Confabulation on IR	0.07	0.14*	1.13	Confabulation DR	0.07	0.12	1.12
NO1	−0.08	−0.34**	2.35	NO2	−0.07	−0.26**	2.05
DE1	−0.07	−0.36**	2.64	DE2	−0.04	−0.22*	2.50
DK1	−0.06	−0.20*	1.69	DK2	−0.04	−0.18*	1.64
GCS	−0.02	−0.13*	1.07	GCS	−0.04	−0.19*	1.21
MDS	0.01	0.20**	1.28	MDS	0.01	0.24**	1.25
SSMQ	0.01	−0.11	1.17	SSMQ	0.01	0.19*	1.26
				Shift	−0.03	−0.08	1.44
	*R*^2^ = 0.264 Δ*R*^2^ = 0.155*** F = 6.415***				*R*^2^ = 0.238 Δ*R*^2^ = 0.120*** F = 5.064***		

*p < 0.05; **p < 0.01; ***p < 0.001. Sex: 1, male; 2, female; NO 1 and 2, simple No answers; DE 1 and 2, Direct Explanation answers; DK 1 and 2, Do not Know answers GCS, Gudjonsson Compliance Scale; MDS, Memory Distrust Scale, SSMQ, Squire Subjective Memory Questionnaire.

## 4 Discussion

The main aim of the present study was to detect whether immediate and delayed suggestibility, measured by the Gudjonsson Suggestibility Scale (GSS 2), are more strongly associated with trait commission errors in memory distrust than omission errors as measured by the MSD and the SSMQ, respectively.

Although both the MDS and the SSMQ assess subjective aspects of memory, they tap distinct metacognitive processes. The SSMQ indexes self-perceived memory ability, whereas the MDS captures the externalisation of memory judgement, a process directly implicated in the acceptance of misleading post-event information. Including both measures in the present study allowed the authors to test whether interrogative suggestibility is more strongly associated with commission-related memory distrust than with general subjective memory complaints, thereby clarifying the specificity of subjective memory factors linked to suggestibility. In this respect, the study adopts a theoretically distinctive and integrative approach.

The hypotheses were supported, apart from Hypothesis 6 where no significant correlation was found between compliance and the MDS and the SSMQ scores. This was a surprising finding in view of previous research (Nash et al., 2023; Van Bergen et al., 2009, 2010a) but may be due to the multifaceted cognitive, emotional and behavioural aspects likely to be involved in the relationship between compliance and memory distrust (see Gudjonsson, 2018, Table 4.1, p. 79).

In the current study, as in the Nash et al. (2023) study the MDS and the SSMQ were significantly correlated suggesting that they are based on similar underlying psychological constructs (i.e., memory distrust). Whilst both measures focus on memory distrust, the SSMQ, which has been more extensively researched than the MDS, focuses on omission errors (i.e., failure to recall information). In contrast, the MDS measures commission errors, which are more relevant to interrogation and false confession (Gudjonsson, 2018). What the two scales have in common is lack of confidence and trust in their memory, which may cause failure to produce memory (omission errors) as well as susceptibility to misinformation (commission errors).

In the current study, as predicted, the MDS and the SSMQ correlated significantly with both immediate and delayed recall with similar effect sizes. The fact that both the MDS and the SSMQ are significantly associated with poor verbal recall on the GSS 2 narrative is important because it shows where the two measures overlap. The shared association establishes *construct validity* for both scales: they are meaningfully connected to actual memory performance rather than being purely subjective or artefactual.

In contrast to verbal memory recall, confabulation on both immediate and delayed recall was significantly correlated only with the MDS, as predicted, highlighting a clear point of divergence between the two measures. This finding is theoretically important because confabulation, as indexed by the GSS, reflects *internally generated commission errors* that are not directly assessed by the SSMQ. In legal contexts, confabulation represents the outcome of an internally generated process of memory distortion, which may be provoked or amplified by specific internal states or external situational triggers, including uncertainty, pressure to respond, or authoritative questioning (Gudjonsson et al., 2021).

In contrast to confabulation, delayed suggestibility on the GSS measures the extent to which misinformation—specifically content derived from earlier misleading questions—is subsequently incorporated into memory recollection at a follow-up session (Vagni et al., 2015, 2017, 2018). It represents a particularly important form of commission error, one that is commonly observed in cases of internalised false confessions (Gudjonsson, 2003, 2018). As predicted, delayed suggestibility was significantly correlated with the MDS, but not with the SSMQ, with a medium effect size. This finding highlights a further point of divergence between the two measures and is of both theoretical and practical importance.

The Memory Distrust Scale (MDS) correlated significantly with Yield 1 and Yield 2, with medium effect sizes, but did not correlate with Shift. This pattern supports Van Bergen’s (2011) proposal that Yield is primarily driven by *trait memory distrust*, whereas Shift reflects *state memory distrust*. The findings therefore indicate that trait-based questionnaires of memory distrust are more strongly associated with Yield responses on the Gudjonsson Suggestibility Scale than with Shift. State memory distrust is situational, transient, and dependent on immediate social pressure, whereas trait memory distrust is enduring, internally driven, and generalised across contexts. Accordingly, Yield responses may index vulnerability to internalised misinformation, whereas Shift responses more closely reflect situational acquiescence under social pressure.

In contrast the SSMQ correlated significantly (small effect size) with Shift and not with Yield 1 or Yield 2. One explanation may be found within the experimental study of Van Bergen et al. (2008), which showed that out of five different interrogation techniques, suggesting to participants that they genuinely may not remember looking at the confidential examination paper due to memory problems had the largest impact on the subsequent SSMQ memory distrust score. However, the GSS 2 instructions in the current study, which included negative feedback, did not have significant impact on the SSMQ score at 1 week follow-up, which suggests that the impact of the Shift administration on memory distrust is temporary. This is a novel finding.

Compliance, as measured by the Gudjonsson Compliance Scale, shows a consistent but partial association with immediate interrogative suggestibility. Empirical studies indicate that compliance is more strongly related to the Shift component of suggestibility—reflecting a tendency to change answers following negative feedback—than to Yield 1, which is more closely linked to memory uncertainty and source-monitoring difficulties (Gudjonsson, 2003, 2018). However, in the present study, compliance correlated most strongly with Yield 2 (medium effect size). This finding is theoretically coherent, as Yield 2 reflects a tendency to acquiesce to misleading questions following the application of social pressure in the form of negative feedback.

In contrast to immediate suggestibility, delayed suggestibility did not correlate significantly with the GCS score. This is theoretically and practically important because it draws a clear boundary between *social–motivational compliance* and *memory-based internalisation of misinformation*—a distinction that sits at the very core of modern false-confession theory (Kassin and Wrightsman, 1985; Gudjonsson, 2018, 2021).

The linear regression models in Table 2 show that the best predictors of Yield 1 and Yield 2 after adjusting for age, sex, immediate recall, and confabulation, recall, were GCS compliance and a high MDS score, adding significantly to the variance in Yield 1 and Yield 2. This suggests that compliance and memory trust concerning commission errors combined are salient components of yielding to leading questions before and after negative feedback after adjusting for memory recall. This supports the suggestion of Mastroberardino and Marucci (2013) that GSS Yield answers include both commission errors (referred to as ‘internalisation of suggested materials’) and compliance. In contrast, supported by the current findings, Shift is more driven by combination of poor recall, memory distrust involving omission errors, and compliance.

Regarding delayed suggestibility, it was the combination of poor recall, type of RBRs (mainly DE and NO answers), compliance, and commission memory trust that contributed to delayed suggestibility prior to negative feedback. The findings after negative feedback were similar, but less marked (23.8% versus 26.4% of the total variance in delayed suggestibility). Trait omission memory distrust (SSMQ) now had significant loading in addition to trait commission memory distrust (MDS). This is a novel finding suggesting that after negative feedback on the GSS 2, trait omission errors become impactful on subsequent delayed suggestibility whereas before negative feedback they were dormant.

It is important to draw a distinction between memory distrust (i.e., dismissing one’s memory recall as a reliable source of information, which can on occasions be profound, leading to internalised false confession; Gudjonsson, 2018) and memory confidence (i.e., uncertainties in memory recall, which may be more transient and less pervasive and impactful). Both are relevant to susceptibility to misinformation in memory (van Bergen et al., 2010b). Lack of confidence in memory and memory distrust fall within the domain of metamemory, which is relevant to both the MDS and the SSMQ (Nash et al., 2023).

Lack of confidence in memory is related to ‘Do not Know’ replies to the leading questions, which is over-represented in people with attention deficit hyperactivity disorder (ADHD; Gudjonsson et al., 2007; Gudjonsson and Young, 2021). In contrast ‘direct explanation’ answers provide evidence of confidence and trust in memory recollection, which is supported by the negative correlation with the MDS.

To the best of our knowledge, this is the first study to show a significant relationship between trait memory distrust and ‘direct explanation’ answers. ‘Direct explanation’ answers reflect successful source discrimination and demonstrates that the interviewee:

- Identifies the suggested detail as *externally generated* (by the interviewer).
- Correctly rejects it as not belonging to the original event.
- Articulates *why* it is incorrect.

This goes beyond simple rejection (“No”) and indicates explicit metacognitive insight into the source of error, which is the essence of effective source monitoring (Johnson et al., 1993).

The main strength of the current study is that the sample was reasonably large, all current measures included in GSS 2 were analysed (Gudjonsson et al., 2024), which to the best of our knowledge provides the first comprehensive study of the relationship between immediate and delayed suggestibility and trait memory trust concerning both omission and commission errors.

The strength of the GSS 2 lies in the fact that its core components—verbal recall, confabulation, immediate suggestibility, delayed suggestibility, and types of resistant behavioural responses—are all derived from observable, performance-based, and objectively scorable indices. These measures are grounded in participants’ actual responses to standardised stimuli and interview conditions, providing direct behavioural evidence of memory performance, suggestibility, and resistance to social pressure.

In contrast, the Gudjonsson Compliance Scale (GCS) is based on self-reported past behaviour, indexing tendencies such as eagerness to please and avoidance of conflict or confrontation. Unlike interrogative suggestibility, which is elicited and measured within a controlled interview context, such retrospective self-reports are potentially susceptible to impression management and malingering, particularly in forensic settings (Gudjonsson, 2018).

The Memory Distrust Scale (MDS) and the Squire Subjective Memory Questionnaire (SSMQ) occupy an intermediate position. Although they measure different aspects of memory self-evaluation, they overlap in several core respects. Both assess subjective beliefs and confidence about memory functioning, rather than objective memory capacity; both capture memory-related vulnerability processes; and both demonstrate adequate psychometric reliability. Their value lies in indexing enduring self-perceptions that may shape behaviour under uncertainty and social pressure.

Critically, however, their robustness differs by function rather than by psychometric quality. The SSMQ is robust as a general trait measure of memory confidence, primarily reflecting omission-based vulnerabilities such as forgetting and uncertainty. The MDS, by contrast, is robust as a forensic-diagnostic vulnerability measure, capturing trait-level memory distrust that is closely linked to commission errors, confabulation, and both immediate and delayed suggestibility.

Taken together, these measures are complementary: the GSS 2 provides objective behavioural indices of suggestibility and resistance, while the MDS and SSMQ clarify the subjective memory beliefs that may predispose individuals to different forms of interrogative vulnerability.

The present study has important forensic implications, as it advances a more holistic and sophisticated framework for evaluating the cumulative impact of multiple, measurable psychological variables when assessing risk factors for unreliable witness and suspect testimony. Rather than relying on single indicators in isolation, the findings highlight the value of examining how distinct but related vulnerability factors interact to influence confession evidence and other forms of forensic testimony.

For professionals working in forensic and legal settings, these variables can be taken into account to better understand their combined impact on confessions, witness statements, and forensic evaluations. There is a strong and continuing need for the development and validation of assessment tools specifically designed for use with witnesses and suspects, in order to promote fairness, transparency, and justice within investigative and judicial processes.

The present study demonstrates the construct validity of both the Memory Distrust Scale (MDS) and the Squire Subjective Memory Questionnaire (SSMQ), and highlights the added value of combining their use. When used alongside immediate and delayed interrogative suggestibility, compliance, and different forms of resistant behavioural responses, these measures provide a more comprehensive and nuanced assessment of psychological vulnerability relevant to the evaluation of confession evidence.

Regarding limitations, the primary focus was on trait memory distrust. State memory distrust on the GSS 2 could also be tested in future studies, using a similar methodology to that of Gudjonsson (1983). This could provide a more complete interaction model of the salient variables, especially in relation to the impact of negative feedback on state trust and delayed suggestibility (Tata and Gudjonsson, 1990).

## Data Availability

The raw data supporting the conclusions of this article will be made available by the authors, without undue reservation.

## Footnotes

1 The MDS and the SSMQ differ in scoring direction because they are anchored in conceptually opposite aspects of memory self-evaluation. The MDS is designed to measure memory distrust directly, with higher scores reflecting greater distrust, whereas the SSMQ measures subjective memory confidence, with lower scores indicating poorer perceived memory functioning. Consequently, elevated memory distrust is reflected by higher MDS scores and lower SSMQ scores.
