# Supplementary material for: How do omission and commission errors of trait memory distrust relate to immediate and delayed suggestibility as measured by the Gudjonsson Suggestibility Scale?
Source: Front Psychol. 2026 Mar 9;17:1774327. doi: 10.3389/fpsyg.2026.1774327 (PMC13006298; doi:10.3389/fpsyg.2026.1774327)
Supplement: Supplementary file 1 [file Appendix_1.docx]

Appendix

A1 - descriptive statistics ( N = 229)

|  | Mean | SD | MIN | MAX |
| --- | --- | --- | --- | --- |
| GSS2: |  |  |  |  |
| Immediate Recall | 18.44 | 4.93 | 9 | 32 |
| Confabulation on IR | 1.32 | 1.47 | 0 | 6 |
| Delayed Recall | 14.73 | 5.46 | 3 | 29 |
| Confabulation on DR | 1.44 | 1.38 | 0 | 6 |
| Yield 1 | 3.84 | 3.06 | 0 | 14 |
| Yield 2 | 4.87 | 3.69 | 0 | 15 |
| Shift | 4.28 | 2.45 | 0 | 10 |
| Total Suggestibility | 8.12 | 4.29 | 0 | 22 |
| Delayed Suggestibility | .51 | .77 | 0 | 3 |
| RBRs: |  |  |  |  |
| NO 1 | 4.98 | 3.13 | 0 | 14 |
| DE 1 | 3.72 | 4.02 | 0 | 15 |
| DK1 | 2.40 | 2.48 | 0 | 12 |
| NO 2 | 4.34 | 2.94 | 0 | 14 |
| DE 2 | 3.44 | 3.94 | 0 | 14 |
| DK 2 | 2.30 | 2.80 | 0 | 13 |
| GCS | 9.80 | 4.28 | 2 | 19 |
| MDS | 56.14 | 17.61 | 25 | 120 |
| SSMQ | 21.09 | 16.77 | -36 | 54 |

A2 – Memory Distrust Scale Italian Version

| English version | Italian version |
| --- | --- |
| 1. I often look for physical evidence, such as photographs, to check whether things really happened the way I remember them. | 1. Spesso cerco prove materiali, come fotografie, per verificare se le cose sono realmente accadute nel modo in cui le ricordo |
| 1. I often turn to other people to help me decide whether my memories are accurate. | 1. Mi rivolgo spesso ad altre persone per aiutarmi a decidere se i miei ricordi sono accurati |
| 1. I tend to question my memories of past events if other people do not corroborate what I remember. | 1. Tendo a mettere in discussione i miei ricordi di eventi passati se altre persone non confermano ciò che ricordo. |
| 1. Sometimes I distrust my own memories if I cannot find any physical evidence to confirm what I remember. | 1. A volte diffido dei miei ricordi se non riesco a trovare alcuna prova materiale per confermare ciò che ricordo. |
| 1. I often have difficulty distinguishing events I remember from those I only imagined. | 1. Spesso ho difficoltà a distinguere gli eventi che ricordo da quelli che ho solo immaginato. |
| 1. I am often unsure whether something that I recall genuinely happened, or whether I only thought or dreamed about it. | 1. Spesso non sono sicuro se qualcosa che ricordo sia realmente accaduto o se l'abbia solo pensato o sognato. |
| 1. I believe some of my memories may have originated entirely from my imagination. | 1. Credo che alcuni dei miei ricordi possano aver avuto origine interamente dalla mia immaginazione. |
| 1. I am sometimes uncertain whether an event that I recall really happened to me, or whether I saw it on TV or in a movie. | 1. A volte sono incerto se un evento che ricordo mi sia realmente accaduto o se l'abbia visto in TV o in un film. |
| 1. Other people sometimes describe past events in ways that make me doubt my own recollection of those events. | 1. Altre persone a volte descrivono eventi passati in modi che mi fanno dubitare del mio ricordo di quegli eventi. |
| 1. I could be easily persuaded that an event I remember is impossible. | 1. Potrei essere facilmente convinto che un evento che ricordo sia impossibile. |
| 1. If another person contradicts my recollection of the past, they are probably correct. | 1. Se un'altra persona contraddice i miei ricordi del passato, probabilmente ha ragione. |
| 1. Under the right circumstances, I could be persuaded that any one of my memories was completely false. | 1. Nelle giuste circostanze, potrei essere convinto che qualcuno dei miei ricordi sia completamente falso. |
| 1. I generally have more trust in other people’s recollections of events than in my own recollections. | 1. Generalmente ho più fiducia nei ricordi degli altri che nei miei. |
| 1. I often trust other people’s descriptions of a past event, even if I have a very different recollection of what happened. | 1. Spesso mi fido delle descrizioni degli altri sugli eventi passati, anche se ho un ricordo molto diverso di quello che è successo |
| 1. Other people’s memories are usually more accurate than my own memories. | 1. I ricordi delle altre persone sono solitamente più accurati dei miei |
| 1. My memories are rarely a very accurate reflection of what truly occurred. | 1. I miei ricordi raramente riflettono molto accuratamente ciò che è veramente accaduto. |
| 1. My memories of past events are unreliable. | 1. I miei ricordi di eventi passati sono inaffidabili |
| 1. I cannot always be confident that my memories accurately reflect what really happened. | 1. 18. Non posso sempre essere sicuro che i miei ricordi riflettano accuratamente ciò che è realmente accaduto. |
| 1. I have little trust that many of the events I remember did really occur. | 1. 19. Ho poca fiducia sul fatto che molti dei miei ricordi siano realmente accaduti |
| 1. I sometimes distrust that certain experiences I remember really happened at all. | 1. 20. A volte dubito che certe esperienze che ricordo siano realmente accadute |

A3 – Squire Subjective Memory Questionnaire

| English version | Italian version |
| --- | --- |
| 1. My ability to search through my mind and recall names or memories I know are there is | 1. La mia capacità di richiamare alla mente nomi o ricordi che so che esistono è |
| 2. I think my relatives and acquaintances now judge my memory to be | 2. Penso che i miei familiari e amici giudichino la mia memoria essere come |
| 3. My ability to recall things when I really try is | 3. La mia capacità di ricordare le cose quando mi sforzo è |
| 4. My ability to hold in my memory things I have learned is | 4. La mia capacità di mantenere nella memoria le cose che ho imparato è |
| 5. If I were asked about it a month from now, my ability to remember facts about this form I am filling out would be | 5. Se tra un mese mi chiedessero di ricordare le informazioni contenute in questo modulo la mia capacità sarebbe |
| 6. My ability to make a past memory that is ‘on the tip of my tongue’ available is | 6. La mia capacità di dire un ricordo del passato che è "sulla punta della mia lingua" è |
| 7. My ability to recall things that happened a long time ago is | 7. La mia capacità di ricordare cose accadute molto tempo fa è |
| 8. My ability to remember the names and faces of people I meet is | 8. La mia capacità di ricordare i nomi e i volti delle persone che incontro è |
| 9. My ability to remember what I was doing after I have taken my mind off it for a few minutes is | 9. Dopo che mi distraggo per alcuni minuti, la mia capacità di ricordare cosa stavo facendo è |
| 10. My ability to remember things that have happened more than a year ago is | 10. La mia capacità di ricordare cose accadute più di un anno fa è |
| 11. My ability now to remember what I read and what I watch on television is | 11. In questo periodo la mia capacità di ricordare cosa leggo e cosa guardo in televisione è |
| 12. My ability to recall things that happened during my childhood is | 12. La mia capacità di ricordare cose accadute quando ero piccolo è |
| 13. My ability to know when the things I am paying attention to are going to stick in my memory is | 13. La mia capacità di sapere che le cose a cui sto dando attenzione rimarranno impresse nella mia memoria è |
| 14. My ability to make sense out of what people explain to me is | 14. La mia capacità di dare un senso a ciò che gli altri mi spiegano è |
| 15. My ability to reach back in my memory and recall what happened a few minutes ago is | 15. La mia capacità di tornare indietro nella memoria e ricordare cosa è successo pochi minuti fa è |
| 16. My ability to pay attention to what goes on around me is | 16. La mia capacità di prestare attenzione a ciò che accade intorno a me è |
| 17. My general alertness to things happening around me is | 17. In generale la mia prontezza nel cogliere le cose che accadono intorno a me è |
| 18. My ability to follow what people are saying is | 18. La mia capacità di seguire ciò che gli altri dicono è |
